# Two-Aperture Microfluidic Probes as Flow Dipole: Theory and Applications

**DOI:** 10.1038/srep11943

**Published:** 2015-07-14

**Authors:** Mohammadali Safavieh, Mohammad A. Qasaimeh, Ali Vakil, David Juncker, Thomas Gervais

**Affiliations:** 1 Institut national de la recherche scientifique (INRS)- Énergie Matériaux Télécommunications (EMT), Université du Québec, 1650 Boulevard Lionel-Boulet, Varennes, J3× 1S2, Québec, Canada; 2 Department of Biomedical Engineering, McGill University, Montréal, Quebec, H3A 1A4, Canada; 3 Division of Engineering, New York University Abu Dhabi, Abu Dhabi, United Arab Emirates; 4 Department of Mathematics, University of British Columbia, Vancouver, British Columbia, V6T 1Z2, Canada; 5 Department of Neurology and Neurosurgery, McGill University, Montréal, Quebec, H3A 1A4, Canada; 6 McGill University and Genome Quebec Innovation Centre, Montreal, Quebec, H3A 1A4, Canada; 7 Department of Engineering Physics, Polytechnique Montréal, Montreal, Quebec, H3C 3A7, Canada; 8 Centre de recherche du Centre hospitalier de l’Université de Montréal, and Institut du cancer de Montréal, Montreal, Quebec, H2L 4M1, Canada

## Abstract

A microfluidic probe (MFP) is a mobile channel-less microfluidic system under which a fluid is injected from an aperture into an open space, hydrodynamically confined by a surrounding fluid, and entirely re-aspirated into a second aperture. Various MFPs have been developed, and have been used for applications ranging from surface patterning of photoresists to local perfusion of organotypic tissue slices. However, the hydrodynamic and mass transfer properties of the flow under the MFP have not been analyzed, and the flow parameters are adjusted empirically. Here, we present an analytical model describing the key transport properties in MFP operation, including the dimensions of the hydrodynamic flow confinement (HFC) area, diffusion broadening, and shear stress as a function of: (i) probe geometry (ii) aspiration-to-injection flow rate ratio (iii) gap between MFP and substrate and (iv) reagent diffusivity. Analytical results and scaling laws were validated against numerical simulations and experimental results from published data. These results will be useful to guide future MFP design and operation, notably to control the MFP “brush stroke” while preserving shear-sensitive cells and tissues.

Microfluidics refers to the manipulation of minute amounts of liquids, which typically takes place within the confinement of closed microchannels^1^,^2^,^3^. However, one of the drawbacks of “closed” microfluidics is the challenge to process objects likely to induce clogging or that are simply too large to fit in a channel network, such as tissue sections, large cells and particles. Open microfluidic systems overcome some of these problems by using hydrodynamic flow confinement (HFC) to confine a fluid stream such as in microfluidic probes (MFP)^4^,^5^,^6^ or capillary effects to confine droplets between a probe tip and a surface, such as in the “chemistrode” configuration^10^,^7^,^8^. Using the HFC property of MFPs, reagents injected by the probe can be confined within close proximity of the probe, allowing their delivery to a surface with high spatial resolution and lower shear stress than in channel-based microfluidics systems.

In its most general definition, a MFP is an open microfluidic system consisting of a flat, blunt tip with two apertures for injection and re-aspiration of a liquid stream in a gap between the probe and a substrate while hydrodynamically confining it with the surrounding fluid. Fig. 1 shows a schematic of a two-aperture MFP in operation. Reported MFP applications include surface gradients generation^9^, high resolution additive and subtractive bio-patterning of biomolecules and single cells in a highly-controlled fashion^4^, maskless lithography by dissolving photoresist on a coated surface^10^, lysis of human breast cancer cells and collecting and analysis of mRNA released from the lysate^11^, pharmacokinetic studies on cells^12^, staining of tissue sections^13^,analysis of neutrophil dynamics during chemotaxis^14^,^15^, and perfusion of organotypic brain slices^16^. In all these studies^17^, the MFP design, flow rates and gap height were optimized empirically and calibrated experimentally based on a trial and error method. Previous analyses in other probe geometries (microfluidic quadrupoles) have demonstrated that the steadiness and high reproducibility of MFP patterns are due to the presence of Hele-Shaw flow patterns at sufficiently small gap sizes. Under these conditions, the flow underneath the probe can be considered quasi two-dimensional and readily analyzed using potential flow theory drawing upon the strong analogy between 2D flow fields and electrostatic fields^9^. Christ and Turner have studied convection in various two-aperture MFP configurations^18^. Their hydrodynamic pressure measurements at several points underneath the probe, supported by numerical models and relevant pressure scales thoroughly confirm the validity of potential flow assumptions when modeling the flow behavior under the probe. Yet, their analysis does not provide an analytical framework to quantify neither advection nor diffusion in two-aperture MFPs. Advection and diffusion are essential parameters to operating MFPs and control diffusion gradient generation, shear stress at the surface, confinement area, and the effect of port geometry.

Here, we introduce the relevant physical theory describing the various design and operation parameters within a MFP. The two-aperture probe is analyzed as a flow dipole (or doublet)^19^ using the same formalism as for 2D electrostatic dipoles. The model also goes further than the conventional Hele-Shaw formalism by also taking into account diffusive mass transfer. We extract several analytical results as well as scaling laws to control important parameters such as HFC zone dimensions, shear stress and diffusion gradients within the gap below the mesa. The resulting model was used to establish universal rules for systematic probe design and operation. The validity of the analysis was tested against numerical models and experimental data. Finally, the shear stress on the substrate was calculated for various operating conditions and a working range based on the tolerance of cells to shear stress was established.

## Results

**Figure 1:**
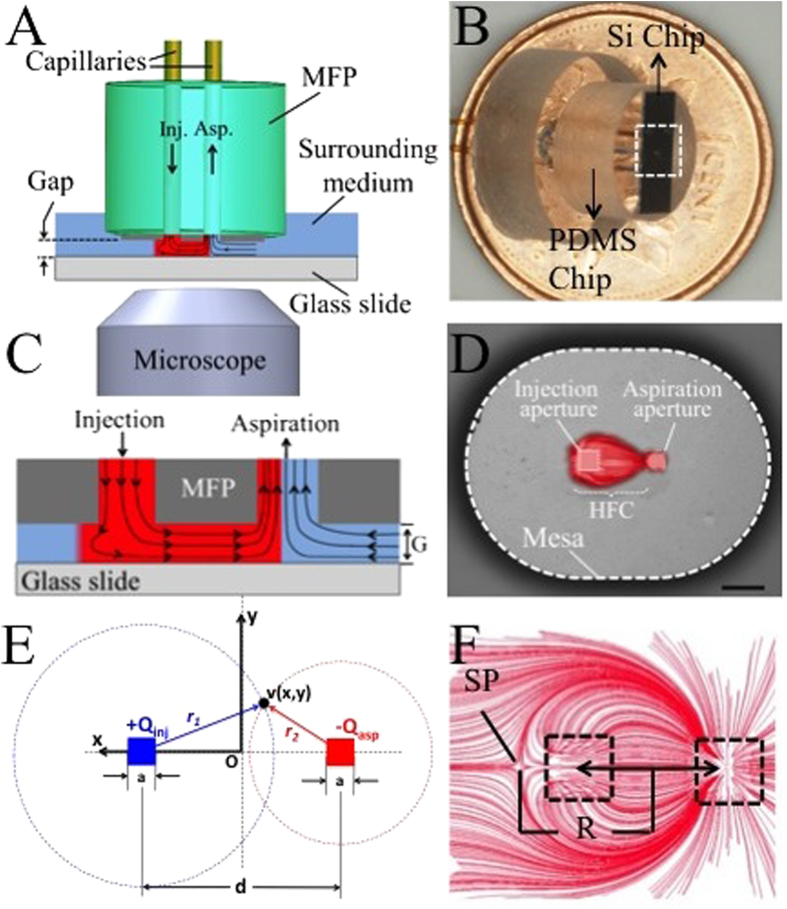
Schematic representation of a two-aperture MFP. (**A**) The MFP is aligned by using the probe holder (not shown here) and is set close to the transparent substrate at the top of the inverted microscope^20^. The fluid is injected and aspirated from the capillaries at the top, which makes the injected flow hydrodynamically confined. (**B**) Picture of the MFP^21^ composed of a silicon chip bonded to a PDMS block with inserted capillaries. The mesa and the microfluidic apertures are enclosed in the white dashed box. (**C**) A close up schematics of the flow under the MFP and the resulting HFC. (**D**) Microscopic image of the MFP showing the mesa and the microfluidic apertures. The scale bar is 50 μm. (**E**) Schematic of a two-aperture MFP for the analysis of flow profiles including a reference frame. The apertures are separated by a distance *d* and are of square side *a*. In the case of round aperture probes, the dimension *a* corresponds to the aperture diameter. The origin was set at the center of the connecting line between the two apertures. (**F**) A finite element model of stream lines under a two-apperture MFP clearly shows the HFC area as well as the stagnation point (SP) located on the far side of the injection aperture. The stagnation position (R) is defined as the distance between the center of the MFP and the SP.

In their most general form, dipolar microfluidic probes comprise two apertures of dimension *a* separated by a distance *d* from center–to-center. The apertures are made into a flat mesa of side dimensions *L*_*1*_ × *L*_*2*_, which is suspended above another flat surface, forming a gap *G* (Fig. 1). Fluid is injected through one of the apertures at a flow rate *Q*_*inj*_ and aspirated back by the second aperture at a flow rate *Q*_*asp*_ = *αQ*_*inj*_, where *α* is a parameter expressing the ratio between aspiration and injection flow rates (>1 for confinement to exist). Detailed experimental procedures for MFP operations are explained elsewhere^4^,^4^.

HFC data described here was obtained from previously published experimental results using a particular MFP geometry (*d* = 50 μm, square apertures with *a* = 25 μm, and mesa dimensions of (550 μm × 447 μm)^4^. The gap size and ratio of injected to aspirated flow rates were changed from one experiment to another.

**Figure 2:**
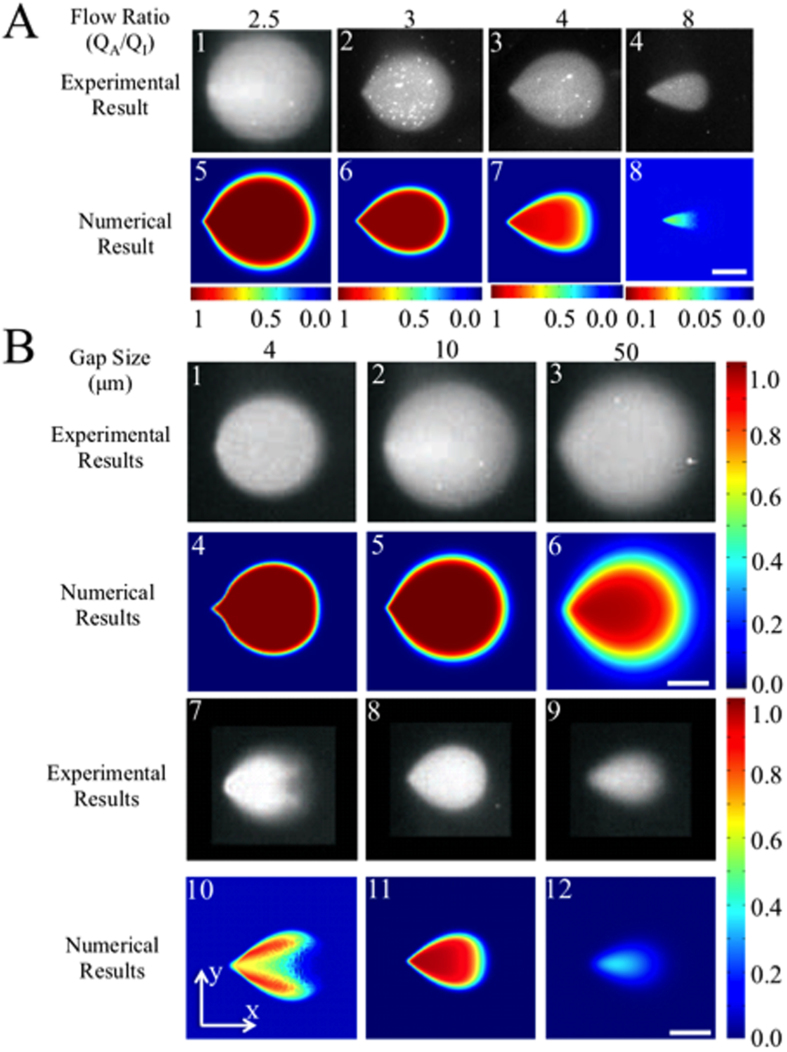
HFC patterns for different flow ratios and gap sizes. (**A**) (1–4) Fluorescence micrographs of the adsorbed protein pattern are shown for various flow ratios. (5–8) Corresponding HFC simulation results for various flow ratios. Gap between MFP and substrate is kept at *G* = 10 μm. The scale bar is 50 μm. (**B**) (1–3, 7–9) Fluorescence micrographs of the adsorbed protein on the surface are shown for different gap sizes. Images are from Juncker, D., Schmid, H. & Delamarche, E. Multipurpose microfluidic probe. *Nat. Mater.* 4, 622–8 (2005). (4–6, 10–12) Numerical simulations show the concentration of injected protein at the bottom substrate. The flow ratios are (1–6) 2.5 and (7–12) 4. Scale bar : 50 μm. In all experiments and simulations, the injection flow rate was set at *Q*_*inj*_ = 0.44 nL/s, and the IgG diffusion was set to *D* = 40 μm^2^/s.

The size of the HFC was determined experimentally by injecting a solute, namely fluorescently labelled IgG molecules under the MFP, which was atop a substrate where the IgGs readily adsorbed. The size of the adsorption area following a 10 s exposure was determined by fluorescent imaging as illustrated in Fig. 2.

Leakage-free HFC was achieved for flow ratios *α* ≥ 2.5 for the particular MFP described in Fig. 2. For lower values of *α*, the injected fluid flow pattern is sufficiently large to extend into the area outside the mesa and is no longer confined. The binding adsorption patterns of IgGs on the substrate were controlled by the flow ratio *α* and strongest for flow ratios between *α* = 2.5 to *α* = 4. For increasing flow ratios, the surface patterns gradually vanished, indicating that the injected IgGs were instead recaptured by the aspiration inlet without reaching the substrate surface.

The confinement as a function of the gap between the mesa of the MFP and the substrate was also assessed experimentally (Fig. 2B). For gaps varying from 4 μm to 50 μm and for flow ratios of 2.5 to 4, the HFC is relatively insensitive to the gap size. However, for smaller gap sizes and large flow ratios, a “wing effect” can occur, by which the diffusion gradient is located underneath the injection aperture (See Figs. 2B(7) and 2B(10)). For a flow ratio of 4, the size of the adsorbed pattern shrinks with increasing gap size. At large enough *α* values, the adsorption pattern disappeared completely when the gap became too large. Thus for a range of flow ratios, the surface pattern shrinks for increasing gap sizes, but at the same time the intensity of the fluorescence weakens reflecting that the local concentration of the molecules in solution near the substrate diminishes as the MFP is moved further away (Fig. 2B). Quantitative criteria have been derived, based on the theory presented below, to determine the critical values of *α and G* under which the adsorption pattern is stable (see Supplementary Information).

### Theory: Formulation of the general MFP problem

The general configuration describing flow in a gap of height *G* right below a MFP is introduced schematically in Fig. 1E. When the gap *G* between the plates is sufficiently small, the flow profile between the plates can be described as a quasi two-dimensional flow, or Hele-Shaw flow^22^,^23^ (See supplementary information), mathematically akin to Darcy’s law^24^.


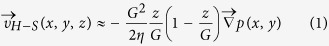


with z being the vertical distance above the bottom flat surface inside the probe where the no-slip boundary conditions are satisfied at *z* = 0 and *z* = *G* The low Reynolds number assumption behind the Hele-Shaw flow approximation requires small inertial forces compared to the pressure and viscous forces in the flow. The Reynolds number of the flow underneath the probe is *Re* = *ρUL*/*η*, where the characteristic length *L* = *G*^2^/*d* under the lubrication approximation and *U* is the characteristic velocity found under the probe. From the working conditions yielding the highest Re number in this paper, we obtain *Re*_*max*_ < 0.11 (a detailed derivation is provided in the supplementary information).

In this configuration, the flow exhibits a parabolic flow profile in the direction of local in plane fluid flow. A pseudo two-dimensional flow profile arising from a scalar potential function *p*(*x, y*) can be obtained by defining the height-averaged velocity under the probe^19^.


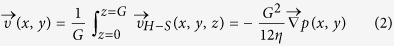


where *η* is the fluid dynamic viscosity and *p*(*x, y*) is the pressure distribution. In open microfluidics, the Hele-Shaw condition is generally met as it requires both creeping flow (Re ≪ 1) and that the flow profile varies smoothly over a distance *L* much larger than the gap *G* (specifically (*L*^2^ ≫ *G*^2^)) where *L* is the characteristic length in (*x, y*) of the observed flow behavior. When Re ≪ 1, the superposition principle can be applied to add up the flow potential of the injection and aspiration apertures to compute a doublet^19^, a flow profile with streamlines mathematically identical to the field lines in a two-dimensional electrostatic dipole, or even more physically accurate, to the current density lines in a two-dimensional current dipole.

### Analysis of the ideal MFP

Unlike electric charges, no flow apertures can be practically reduced to a point source of streamlines. Instead, they must be viewed as openings (round, square, or others) of characteristic dimension *α*. Yet, when the distance between the apertures is much larger than the apertures, the point source approximation is very effective in modeling multipolar flow behavior. We consider here the circular aperture as a general case. Since we are in the presence of a potential flow, Gauss’s theorem^9^ (Eq. (3)) can be readily used to readily calculate in polar coordinates the velocity profile outside a round aperture of thickness *G* and radius *a*/2:


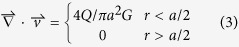


In polar coordinates, the mass balance equation provided above yields the simple result:


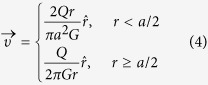


The flow profile under the MFP can be calculated as a superposition of two monopoles of different flow rates located at ±*d*/2 from the origin on the *x*-axis as illustrated in Fig. 1E. When *a* ≪ *d*, the point source approximation is accurate, and the dimension *a* can be neglected. The full velocity profile under the probe is then given in Cartesian coordinates by:


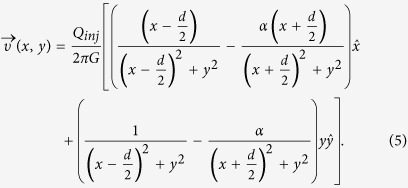


**Stagnation point and hydrodynamic confinement limit**

When *α* > 1, a point of zero velocity (stagnation point) occurs outside the injection aperture at a distance *χ*_*SP*_ of the center of the probe as shown in Fig. 1F. The location of this stagnation point satisfies the condition:


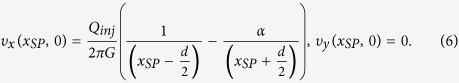


The latter condition on *υ*_*y*_ is satisfied for all *y* due to the symmetry of the problem along the *x*-axis. The condition on *υ*_*x*_ specifies the position of the stagnation point:


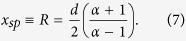


**Figure 3:**
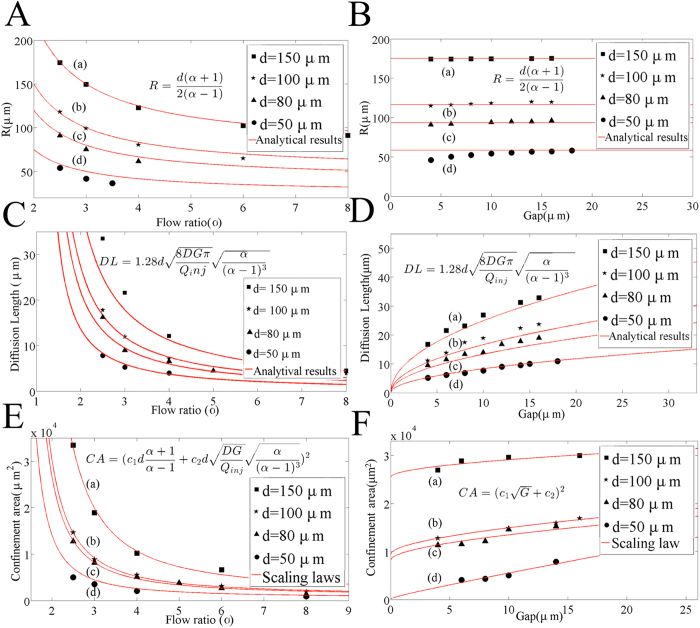
Scaling of confinement area and effect of diffusion broadening on HFC. The set of simulation data (shown in black dots), and scaling laws has been shown in each graphs with (a) *d* = 150 μm (a = 25 μm, round), (b) *d *= 100 μm (a = 25 μm, round), (c) *d* = 80 μm (a = 25 μm, round), (d) *d* = 50 μm (a = 25 μm, square). Mesa sizes were sufficiently large not to induce significant effect on the flow. Effect of (**A**) flow ratio and (**B**) gap size on the stagnation position *R*. The diffusion length varies with (**C**) the flow ratio (~*Q*_*inj*_^−1/2^) and (**D**) the gap size (~*G*^1/2^). (**E**) The confinement area varies as a function of flow rate (**E**) and gap size (**F**) according to the scaling laws in **A**–**D**. The gap size in (**A**) (**C**) (**E**) is 10 μm. The flow ratio in (**B**) (**D**) (**F**) is set to α = 2.5. The default values for injection flow rate was 0.44 nL/s, for the flow ratio was α = 2.5, the gap between the MFP and the substrate was *G* = 10 μm, and for the diffusion coefficient of the injected flow was *D* = 40 μm^2^/s, and were varied one at a time in each experiment. The fit coefficients in (**E**) and (**F**) are shown in Table 1 as well as the coefficient of determination *R*^*2*^ for every model used.

This key result describes the maximum distance *R* from the probe center at which the injected analyte will travel before being recaptured by the aspiration inlet. In other words, injected analytes are confined within closed streamlines as represented in Fig. 1F. The definition of *R* is highly analogous to the confinement length defined earlier for microfluidic quadrupoles, in which 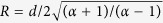
9. In both cases, when *α* = 1, injection and aspiration flow rates are equal and the stagnation point is found at infinity, just as in a regular dipoles and quadrupoles. In Fig. 3A,B, the numerical values of R are plotted against their theoretical value obtained in Eq. (7) as funtions of α and G. Coefficients of determination *R*^2^ are listed in Table 1. As expected, the best match is obtained for the probes where the ratio of aperture size to interaperture distance (a/d) is minimum (R^2^ = 0.99 for a/d = 1/6) and the worst for the largest a/d ratios (R^2^ = 0.96 and 0.92 for a/d = ½). Thus the R^2^ value provided in Table 1 can be viewed as a quality test of the point source analytical model when compared to the full 3D numerical solutions.

An exact expression for the width of the HFC underneath an ideal doublet can also be calculated using a similar method involving stream functions (see supplementary information).

### Scaling laws of diffusion broadening along the HFC area

In most practical situations, analytes within the probe will have a non-negligible diffusivity and will induce a concentration gradient on the outskirts of the HFC area. This broadening will yield significantly larger surface adsorption patterns than expected from the HFC alone. Furthermore, this diffusive broadening can be exploited, as described elsewhere, to generate rapidly tunable, highly reproducible sharp floating concentration gradients^9^. The general advection-diffusion equation of a diffusive species C(*x*, *y*) within under the MFP is described in cartesian coordinates by the equation:


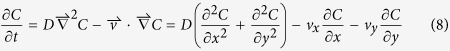


where 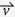 is found in Eq. (5). While easily solved using finite element methods, the unwieldy Eq. (8) offers little physical insight in its current form. However, a scaling law describing the characteristic diffusion length of the HFC can be obtained by linearizing the velocity profile near the stagnation point using a Taylor series expansion of Eq. (5). Therefore:


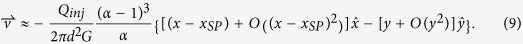


To extract the Péclet number near the stagnation point we make Eq. (8) and Eq. (9) dimensionless, by using the following scales:


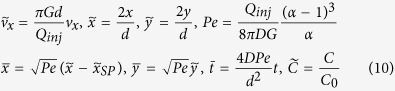


Thus, after simplifications, Eq. (8) can be expressed under its canonic form:


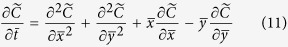


Although Eq. (11) has separable variables, we leave to future work the determination of its full solution, which would need to take into account the curvature of the diffusion interface near the stagnation point, which itself depends on several operation variables. In a first approximation, analogous to the one performed on quadrupoles elsewhere^9^, we make Eq. (11) one dimensional by setting the y-components of the concentration derivatives to 0. In most cases, this approximation is highly justified since the interface radius of curvature is much larger than the diffusion length scale. Furthermore, the diffusion broadening region, in almost all cases in Fig. 2, display a uniform thickness even far from the SP, only decreasing in thickness when nearing the aspiration aperture. The approximation is further justified when using square apertures as they flatten the diffusion profile at the SP even at small curvature radii (e.g. Figs 2A(7) and 2B(11)). Finally, the symmetry plane along the y-axis implies that 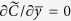 near *y* = 0. When considering the steady state solution only and setting 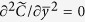, the simplified Eq. (11) with its boundary conditions becomes:


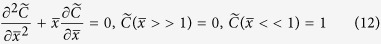


The boundary conditions in Eq. (12) are valid only for high Peclet number, i.e. when the diffusion broadening area is thin an outside of the injection aperture, a condition typically met in probe operation. Solving Eq. (12) yields:


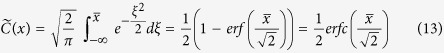


where *erfc(x)* is the complementary error function^25^.

### Practical considerations in MFP operation

The time scale *t*_*0*_ *=* *d*^*2*^*/(4DPe)* is the characteristic time for which the steady state diffusion profile is achieved when the system is perturbed either by a movement of the probe or by a change in the flow rates applied. It is of interest to note that the settling time decreases with an increasing flow ratio α. When α = 1, the settling time becomes theoretically infinite as the HFC zone extends to infinity. The characteristic length scale 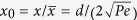 is the characteristic length of the diffusive broadening (concentration gradient) at the edge of the HFC. A diffusion length scale can be experimentally defined as the distance between the points of concentration 0.1 and 0.9. Applying this criterion to Eq. (13) yields:


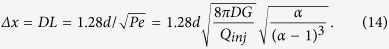


Using typical experimental conditions for MFP operation^7^, *Q*_*inj*_ = 0.44 nL/s, *G* = 10 μm, *d* = 50 μm, *α *= 2.5, *D *= 40 μm^2^/s (IgG), we find the specific values of *Pe *= 60, t_0_ = 260 ms, and *x*_*0*_ = 8 μm. Thus, the settling time is short (<1 s ) increasing linearly as *t*_*0*_ ~ *G* and *t*he diffusion length is fairly steady, increasing as *x*_*0*_ ~ *G*^*1/2*^. Finally, the area of the HFC scales as the square of the characteristic length L_c_^2^ ~ [(*c*_1*_R *+* *c*_2_*Δx*/2*)]^2^, where *c*_*1*_ and *c*_*2*_ are proportionality constants. As both R and Δx linearly depend on d, the area of the HFC scales as *d*^*2*^ (see supplementary information). The validity of the scaling laws derived in the previous section is verified by comparing with full 3D numerical simulations in Fig. 3. Strong agreement was found between the simulation and theory for the stagnation point location, confinement area as well as diffusion length, as described by the *R*^2^ values compiled in Table 1. Calculations of scaling laws for the confinement area has been explained in further details in the supplementary information.

### Shear stress analysis

The MFP has been used to expose cells and tissues with various chemicals, and an important parameter that can influence the treatment outcome is shear stress. Shear stress plays a major role in many biological phenomena bringing about a number of cellular/intracellular events^26^,^27^,^28^. For example, endothelial cells and neutrophils transduce the applied shear stress stimulus into intracellular biochemical responses, by which they regulate the vessel structure^29^,^30^. Similarly, these stimuli also affect the orientation of osteoblasts and neurons resulting in cellular migration and matrix outgrowth^31^,^32^. An important application of the MFP is detaching shear sensitive cells without damaging the cell membrane, and thus calibration of shear stress is necessary.

In Hele-Shaw flows, the shear stress is directly proportional to the normalized velocity profile:


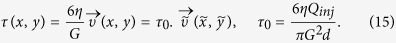


**Figure 4:**
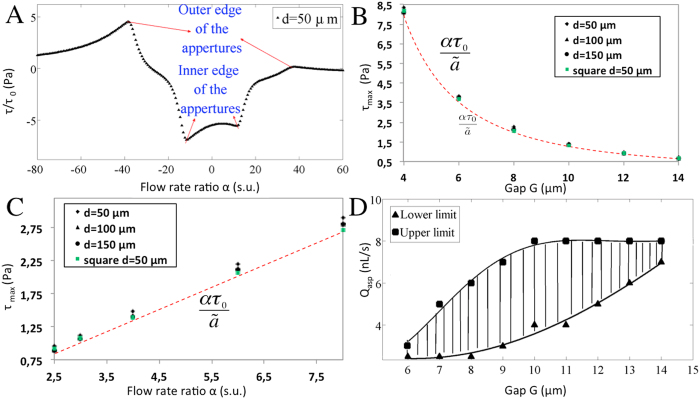
Shear stress distributions under the two apertures MFP. (**A**) Dimensionless shear stress distribution profiles along the middle line at the bottom substrate showing that max shear stress occurs at the inner edge of the aspiration aperture (α = 2.5, *Q*_*inj*_ = 0.44 nL/s). (**B**) Maximum shear stress at the bottom of the substrate as a function of gap size G (α = 4, *Q*_*inj*_ = 0.44 nL/s). The dash line represents the shear stress approximation found by neglecting the contribution of the injection aperture (monopole approximation), which slightly underestimates Eq. (15) by a maximum of 15% when *d/a* > 2 and α > 2. (**C**) Maximum shear stress at the bottom surface as a function of the flow rate ratio α (*G* = 10 μm, *Q*_*inj*_ = 0.44 nL/s). Dash line: monopole approximation of the shear stress. (**D**) Calibration of MFP’s operation conditions with respect to two parameters of flow ratio and gap size to apply shear stress on neurons. The upper and lower limits are 1.5 Pa and 0.5 Pa, respectively. The hatched zone represents appropriate condition of flow ratio and gap size for local cell detachment by trypsinization while avoiding shear-induced damage to the surrounding cells.

The maximum value τ_max_ is found where the maximum value of 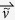 lies, i.e. at (*x* = *−(1* *−* *a/d)*, *y* = *0*) near the inner edge of the aspiration aperture (Fig. 4A). To reflect this observation, we rescale τ_max_ using the aspiration flow rate αQ_inj_ as the new characteristic flow rate and using the aperture size *a* instead of probe length *d* as the new characteristic length. It ensues that when 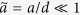 or *α* ≫ 1, the contribution of the injection aperture to the maximum velocity becomes insignificant, giving the simple scales 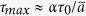 and 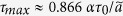 , respectively for the round and square aperture probes for the maximum shear rate under the probe (see supplementary information). Thus maximum shear stress is inversely proportional to gap *G* (~1/G^2^, Fig. 4B) and varies linearly with the aspiration flow rate *Q*_*asp*_. Hence, we expect the maximum shear stress to vary linearly with the flow ratio, as shown in Fig. 4C. Maximal shear stress is an important experimental parameter since in biological systems cells are affected by shear, and for example the growth rate of endothelial cell was shown to be susceptible to shear as low as 1 Pa^33^.

High shear stresses can cause damage to the cell membrane, and consequently, lead to cell death^34^. To account for this issue in MFP design and operation, the maximal shear stress at the bottom of the substrate is characterized first by varying the flow ratio and then by varying the gap size while keeping the other parameter constant. Combining the knowledge from former studies of cell detachment using low shear stress (around 0.5 Pa)^32^ to detach PC12 neural cells and maximum shear stress that can be tolerated by neurons (around 1.5 Pa)^32^, we performed a set of simulations with different gaps and flow rate ratios. By considering both limits described above, we outlined the safe operational parameters of the MFP to work with neurons (Fig. 4D).

### HFC contour and width

Another important application of the current MFP model is to allow precise control over the shape and dimensions of the probe writing head based on user-controlled parameters. When used in surface patterning mode, the dimensions of the HFC area will dictate the shape of the “brush stroke” that the MFP leaves when it writes on a surface. One can proceed to visualize the shape of the hydrodynamic flow confinement (HFC) area by determining the associated stream function^22^ of the velocity profile. In general terms, the stream function is defined as:


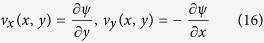


Thus, integrating the y-velocity component in normalized coordinates of Eq. (5) yields:


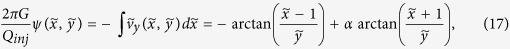


where the integration constant can be set to zero. As the streamlines define the trajectory of an infinitesimal particle in the flow field, the streamline passing by the stagnation point (2*x*_*SP*_*/d* *=* *(α* *+* *1)/(α* *−* *1*) , 0) will describe the envelope of the HFC (neglecting diffusivity). In Eq. (11), the streamline corresponding to this condition is


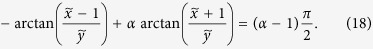


**Figure 5:**
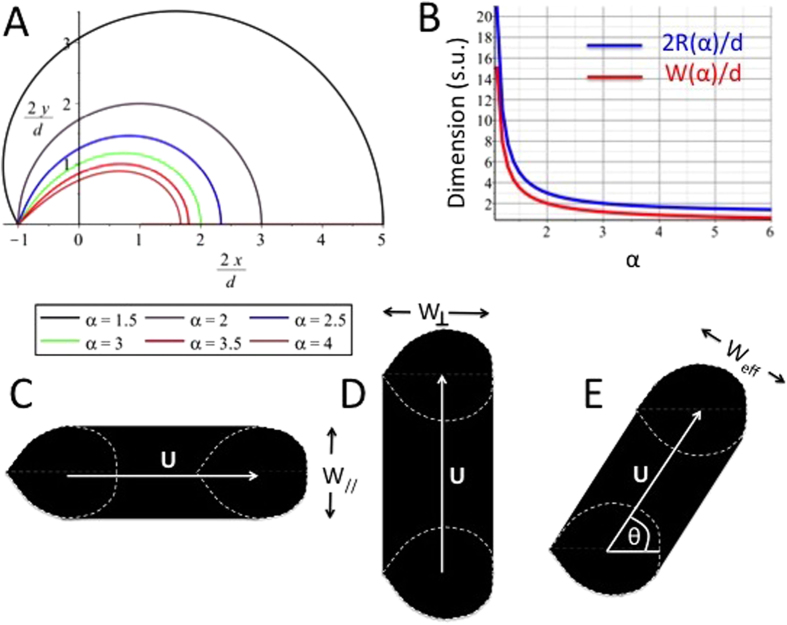
HFC shape and dimension. (**A**) Stream functions representing the contour of the HFC in the symmetrical upper half plane for the ideal point source probe. Plots are presented for various flow ratios *α*. The particular case of *α* = 2 is a half circle of radius identical to the interaperture distance *d*. (**B**) Plot of the normalized half width *W(α)/d* and the normalized length *2R/d* of the HFC with respect to the flow rate ratio α = *Q*_*asp*_*/Q*_*inj*_. Although the width is systematically shorter than the length in all cases, the two dimensions follow the same scale (linear with *d*, and varying as 1/(α − 1)). (**C**)Various brush strokes produced by the moving MFP in surface patterning mode. Movement (**C**) parallel to probe axis, (**D**) perpendicular to probe axis, (**E**) arbitrary at an angle θ with the probe’s axis.

Hence, Eq. (18) is the function describing the upper half (y > 0) of the HFC contour. While the variables cannot be separated, the shape of the implicit stream function is plotted in Fig. 5A forvarious values of *α.*

The HFC area or simply confinement area (C. A.), i.e. the area of the spot left by the probe in surface patterning mode, can be found by plotting Eq. (18) for a specific value of *α* and numerically integrating under the curve. One exception occurs when *α* = 2 where Eq. (18), once simplified, yields (2*x*/*d* − 1)^2^ + (2*y*/*d*)^2^ = 4, which is the equation of a circle of radius *x* = *d* centered at the injection aperture (*x* = *d/2*) (Fig. 5A). The confinement area of the HFC in this particular case is *C.A.* = *πd*^2^. Another exception occurs when *α* = 3, which also has a simple solution: *C.A.* = 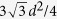 = 1,299 *d*^*2*^. Other cases can be found numerically, for example *C.A.* = 8,712 *d*^*2*^ (α = 1.5), *C.A.* = 0,8264 *d*^*2*^ (α = 4), *C.A.* = 0,6130 *d*^2^ (α = 5), *C.A.* = 0,4908 *d*^*2*^ (α = 6). In all cases, confinement area will be proportional to the square of the inter-aperture dimension *d* and will decrease with increasing flow ratio *α*. Diffusion is set to 0 everywhere in this section to highlight the contribution of the flow profile alone to the C.A.

### Theoretical HFC Width

Perhaps the simplest and most generally applicable result of this paper is the positions of the probe’s stagnation point *R* as described in Eq. (7). Another useful measurement is the HFC maximum width *W*, the maximum extent of the HFC in the dimension perpendicular to the probe’s main axis. The HFC width as a function of flow rate has been studied numerically and experimentally elsewhere^18^, but without the insight provided by a complete mathematical derivation, which is provided here for the ideal case of point sources under the Hele-Shaw flow assumption.

Since the contour of the HFC can be described theoretically by the stream function described in Eq. (18), we can compute the local maximum of the HFC width in the y dimension by differentiating the stream function with respect to *x* such that


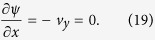


Physically, this represents the point at the edge of the HFC at which the *y* component of the velocity is identically zero. Using the normalized coordinates, we get:


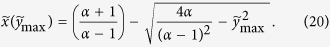


This is the condition that must be respected when *v*_*y*_ = *0* and *y* = *y*_*max*_ = *W/2*, where *W* is the HFC width. Substituting Eq. (20) in Eq. (18) yields:


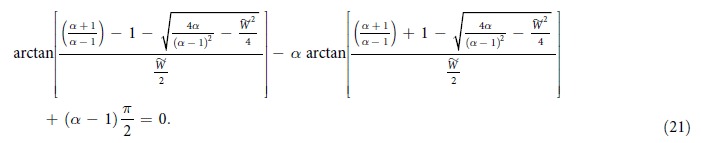


This is the transcendental equation that analytically describes the HFC’s normalized full width (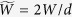). The width function 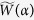 is plotted in Fig. 5B. Note that Eq. (21) is a normalized function and that it is therefore valid for any MFP geometry where the point source approximation can be made.

## Discussion

### Applications to brush stroke analysis and probe operation control

One of the most important applications of the current model is to be able to control the shape and dimensions of the probe writing head by specifying user-defined parameters such as the injection and aspiration flow rates or the probe gap *G*. The contour function described in Eq. (18), combined with the HFC maximum radius *R* (Eq. (7)) and the HFC width W (Eq. (21)) completely defines the area and maximum dimensions of the MFPs writing area. To account for the diffusive broadening around the contour, Eq. (14) can also be applied. However, all these parameters specify the shape of the probe’s writing under static conditions. When the probe is moving at constant velocity *U* in any given direction with respect to a fixed substrate, viscous forces will tend to deform the static flow profile. The fully developed Hele-Shaw profile will be a superposition of a parabolic flow profile stemming from the probe apertures and a Couette flow profile of mean velocity *U/2* induced in the direction of the probe displacement. This flow behavior can be reduced to a purely parabolic flow profile as in Eq. (1) by placing oneself in a referential moving in the direction of the probe at the mean fluid velocity *U*/2. In doing so, the height-averaged flow profile found in Eq. (2) is kept unchanged and the flow in this reference frame is identical to that of Eq. (5) (see proof in supplementary information). Once solved, the velocity profile in the probe’s moving frame, using the dimensionless scales described in Eq. (10), now takes the form


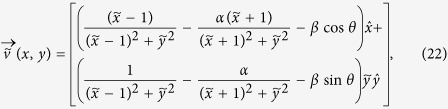


where *θ* is the angle of the probe velocity vector with the probe axis and *β* = *πGUd*/2*Q*_*inj*_ is the dimensionless probe velocity. When β > 1, the probe velocity disturbs the HFC contour significantly. Yet when β ≪ 1, the contour can be assumed not to be perturbed by the probe movement. Using the typical operation parameters previously described (*Q*_*inj*_ = 0.44 nL/s, *G* = 10 μm, *d* = 50 μm), we find that the probe perturbation due to its velocity is negligible when *U* ≪ 2*Q*_*inj*_/*πGd* = 0.6 mm/s. This critical velocity can be made arbitrarily large without changing the HFC contour by increasing *Q*_*inj*_ and *Q*_*asp*_ proportionally while keeping the ratio *α* constant.

Within this approximation of small *β*, we now define a “brush stroke” for the probe operation where the width of the stroke will depend on the user-controlled parameters, including the probe’s velocity and displacement angle (Figs 5(C–E)). The brush width can be decomposed in its two principal axes: the width of the probe when moving parallel and perpendicular to the probe’s axis, respectively 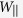
and 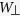, where 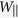 is calculated using Eq. (21) and


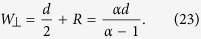


The linear combination describing the brush stroke at low velocities for an arbitrary xy probe displacement is generally described as:


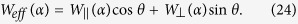


It is important to point out that the diffusion broadening has not been taken into account when deriving Eq. (24) and to do so would require needlessly cumbersome mathematical derivations. However, the effect of diffusion can be simply taken into account by making the approximation that the diffusive broadening is small compared to the HFC radius and increases it uniformly by a distance Δx, obtained from Eq. (14), on each side except near the aspiration aperture. Under this approximation, 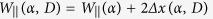
and 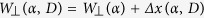 and Eq. (24) can still be used.

## Conclusion

In this paper, we have provided a theoretical analysis of ideal dipolar probes (*d* ≫ *a* ≫ *G*) and tested the validity of our transport model under probes with finite aperture sizes and arbitrary geometries. The analysis is then validated with data obtained from simulations and previously published experimental data. The general set of analytical results and scaling laws are summarized in Table 2. The simple results established will allow precise control of HFC dimensions and confinement area, diffusion length and gradient properties at the HFC’s edges and shear stress control with respect to flow rate ratio *α* and gap size *G*. They will provide precise design criteria (*d*,*a*, and mesa dimensions, during fabrication process) and control the probe’s brush stroke and velocity during operation (*α*, *G*, *Q*_*inj*_, *D*). For example, if the user seeks a circular HFC of area A at the tip, the inter aperture distance *d* to obtain this exact area can readily be calculated (in this special case, when *α* = 2, the HFC area is precisely *A* = *πd*^*2*^, see supplementary information). Once the value of d is found, the dimensions of the mesa need only to be a few times larger than the maximal *R* value of the HFC (with *R* *=* *3d/2* when *α* = 2). Then, using the formula for the HFC outer edge (Table 2), the precise shape of the HFC could then be calculated for any value of *α* for this probe. Finally, the outer edge diffusion layer can be adjusted during the experiment by modulating the gap size *G* while maintaining the HFC’s circular shape constant. In the previous example, we can also determine in advance whether we need square/round, or large/small apertures. Small apertures will be preferred in situations where α is large to prevent the “wing effect” (Fig. 2B-10) that occurs when the stagnation point is found inside the aperture. Large apertures are, on the other hand, preferable when the shear stress needs to be minimal (Fig. 4B,C).

We also provide a complete characterization of the MFP’s “brush stroke” when operating in surface patterning mode. The analysis suggests that the simplest way to precisely control the width of the stroke is to move the MFP either parallel or perpendicular to its axis since the width of the stroke (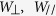) is then completely independent of the flow velocity and given a simple formula (Table 2) that can be easily modified to account for diffusion by adding the diffusion broadening scale (DL, Table 2) to the width.

The results presented in Table 2 can also be used to indirectly measure and calibrate probe height above a surface by looking at diffusion length with respect to G (∝√G) or shear stress as a function of the applied flow rates. Finally, the general theoretical framework provided here, and the set of analytical results that stem from it, enables a more precise control for MFPs of any shape or form in a variety of applications including neuron migration^35^, stem cell differentiation^36^,^37^ and shear-dependent cell poration^38^,^39^ without using costly, complicated trial and error experimental method.

## Methods

### Numerical Modeling

The numerical models were established using the finite element method with commercial software (COMSOL Multiphysics 3.5a, USA) on a computer with 8 cores, 64-bit CPU and 26 GB RAM. The computational model was built by combining Stokes flow and steady state convection-diffusion mass transfer equations. Boundary conditions were assumed to be “no-slip” conditions at solid surfaces and “open boundaries” at atmospheric pressure at the perimeter of the MFP. To compute the mass transfer model, the MFP’s rigid walls and substrate surface are considered as insulators and the flux was set to zero. The concentration at the perimeter was set to zero reflecting the assumption that under HFC no solute reaches the edge, and that the large volume constitutes a sink. Concentration at the injected aperture was normalized and set to 1 to provide a relative scale. At the aspiration aperture, diffusion was neglected owing to the local dominance of convective mass transport.

Numerical calculations of the HFC were performed and the calculated concentration as a function of position at the substrate surface was reported as function of flow ratio in Fig. 2A, and as function of the gap size, Fig. 2B and Fig. 2B. There is a good visual agreement between experimental and numerical results, which was also quantified by measuring the surface area for each case, and comparing it for different flow conditions (Fig. S1). The observed discrepancy between numerical and experimental results can be mainly attributed to the indirect measurement of protein adsorption at the surface rather than the direct measurement of the flow profile. Uncertainty on the experimental gap, exposure time, as well as vibrations of the MFP while running the experiment will cause the protein footprint on the surface to spread. As a consequence, the pattern on the surface is not a record of the average pattern, but of the maximal extent of the flow profile that may occur due to various fluctuations during the 10 s of streaming across the surface. Nevertheless, the numerical models are in good agreement with the adsorbed protein experiments. To extend the validity of our models to any probe geometry and to extract useful design criteria based on scaling laws (see section “Theory”), numerical simulations of the MFP with various geometries and gaps, and under different flow rates were performed using full 3D finite-element models.

To test the limits of our advection/diffusion models, we have proceeded to perform the following series of simulations emulating probe geometries already used in the literature, Table S1. Water was used as fluid (solvent) with a density of 998.2 kg/m^3^ and dynamic viscosity *η* = 0.001 N·m/s (at 20 °C). Immunoglobulin G (IgG) was used as solute and a diffusion coefficient in water of *D* = 4 × 10^−11^ m^2^/s was used^7^. The flow regime was estimated prior to the simulations to help select the appropriate models. The Reynolds number in all regimes was on the order of *Re* ≈ *O*(10^−2^) ≪ 1 indicating creeping flow conditions to be treated using Stokes formalism^22^. Similarly, the Peclet number representing the ratio between convective and diffusive mass transport was found to be high (*Pe* ≫ 1)^40^ for all MFP operation regimes, indicating that convective mass transport dominates diffusion except very close to the stagnation point.

## Additional Information

**How to cite this article**: Safavieh, M. *et al.* Two-Aperture Microfluidic Probes as Flow Dipole : Theory and Applications. *Sci. Rep.* **5**, 11943; doi: 10.1038/srep11943 (2015).

## Supplementary Material

Supplementary Information

## Figures and Tables

**Table 1:** Analytical results and scaling laws for position of stagnation point, confinement area and diffusion length (Fig. 3).

	**Scaling law/analytical Solution**	**a**	**b**	**c**	**d**
A) R *vs* α	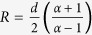	R^2^ = 0.96	R^2^ = 0.97	R^2^ = 0.98	R^2^ = 0.99
B) R *vs* G	idem	*R* = 58.33	*R* = 93.33	*R* = 116.66	*R* = 175
		R^2^ = 0.92	R^2^ = 0.97	R^2^ = 0.98	R^2^ = 0.99
C) DL *vs* α	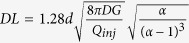	R^2^ = 0.91	R^2^ = 0.96	R^2^ = 0.99	R^2^ = 0.96
D) DL *vs* G	idem	R^2^ = 0.98	R^2^ = 0.95	R^2^ = 0.91	R^2^ = 0.97
E) CA *vs* α	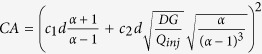	c1 = 0.44	c1 = 0.35	c1 = 0.29	c1 = 0.23
		c2 = 15.43	c2 = 22.77	c2 = 17.71	c2 = 25.46
		R^2^ = 0.99	R^2^ = 0.99	R^2^ = 0.98	R^2^ = 0.99
F) CA *vs* G	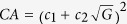	c1 = 121.21	c1 = 244.22	c1 = 284.24	c1 = 471.87
		c2 = 28130	c2 = 10617	c2 = 11900	c2 = 830.31
		R^2^ = 0.92	R^2^ = 0.78	R^2^ = 0.98	R^2^ = 0.98

**Table 2:** Design criteria, formula and validity domain of two apertures MFP. This parameters and related formula provide substantial guide to select best experimental conditions for cell study applications.

**Design Criteria**	**Formula**	**Validity Domain**
HFC outer edge – Eq. (16)	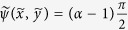	Ideal 2D MFP
Stagnation position (*R*) – Eq. (6)	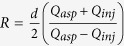	*R* << *L*_*1*_*,L*_*2*_ (mesa dimensions), R ≫ G, d ≫ a
HFC Width parallel to axis – Eq. (20)	*W*_*//*_	Idem
HFC Width perpendicular to axis – Eq. (22)	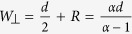	Idem
Péclet Number – Eq. (9)	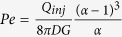	Vicinity of SP
Diffusion length scale (DL) – Eq. (12)	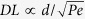	*R *≫ *G*, *d *≫ *a*
HFC Response time – Eq. (9)	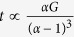	Under Hele-Shaw Condition
Shear stress (τ) – Eq. (14)	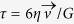	Idem
Max Shear stress	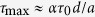	Round apertures
	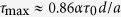	Square apertures
	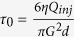	Characteristic scale
