# Erratum: Two-Aperture Microfluidic Probes as Flow Dipoles: Theory and Applications

**DOI:** 10.1038/srep14885

**Published:** 2015-10-08

**Authors:** Mohammadali Safavieh, Mohammad A. Qasaimeh, Ali Vakil, David Juncker, Thomas Gervais

Scientific Reports 5: Article number: 11943; 10.1038/srep11943 published online: 07142015; updated: 10082015.

The original version of this Article contained an error in the title of the paper, where the word “Dipoles” was incorrectly given as “Dipole”.

In addition, there were typographical errors.

In the Results section under subheading ‘Practical considerations in MFP operation’,

“Using typical experimental conditions for MFP operation^7^”

Now reads:

“Using typical experimental conditions for MFP operation^9^”

In reference 33: “Younga” now reads “Young”.

These errors have now been corrected in both the PDF and HTML versions of the Article.

