# Supplementary material for: Two-Aperture Microfluidic Probes as Flow Dipole: Theory and Applications
Source: Sci Rep. 2015 Jul 14;5:11943. doi: 10.1038/srep11943 (PMC4500946; doi:10.1038/srep11943)
Supplement: Supplementary Information [file srep11943-s1.docx]

ELECTRONIC SUPPLEMENTARY INFORMATION

Two-Aperture Microfluidic Probes as Flow Dipoles: Theory and Applications

Mohammadali Safavieh, Mohammad A. Qasaimeh, Ali Vakil, David Juncker and Thomas Gervais

**1. Comparison between experiment and simulation of HFC area for an MFP with two apertures**

| B  A |  |
| --- | --- |

FIG. S1 Comparison between experimental and numerical results in total area of confinement vs. (a) different flow ratios (b) gap sizes between MFP and substrate. (a) Effect of flow ratio on total area of HFC at the bottom substrate. Diffusion coefficient for all cases is 40 μm²/s. The dash lines in each graphs serves as the guide to the eye. By increasing the flow ratio, total area of HFC is getting smaller. The gap between MFP and substrate is 10 μm. (b) Effect of gap size on the HFC. By increasing the gap size, HFC will be larger. The aspiration ratio is 2.5. Numerical results systematically underestimate the experimental observations by roughly 25%. This behavior is to be expected as the experimental confinement area is based on surface adsorption of fluorescent IgG and not directly on the fluorescence intensity in the fluid itself. As time passes by, the adsorption front slowly moves outward.

**2. Calculation of the confinement area**

**
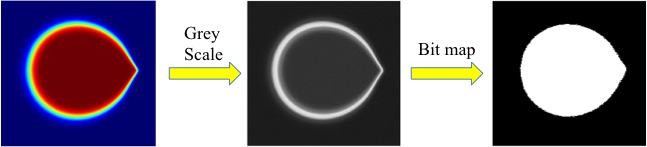
**

FIG S2 Schematic of the image processing for calculating the confinement area in a microfluidic probe with two apertures. The confinement area image is transformed into the grey scale using the COMSOL package. Then by setting the threshold value into 0.5 and transforming into the bitmap image using Adobe Photoshop CS5. The white pixels of the image are added to calculate the area. Using the aperture area as the scale, the confinement area in pixels is converted to a surface area expressed in μm^2^.

**3. Comparison between normalized velocity of round and square opening in MFP**

FIG. S3 Comparison of velocity profiles in square and round aperture MFP (A) Plot of the x component of the normalized velocity profile for 2D dipolar probes with square and round apertures of characteristic dimension *a* ($\alpha$= 2.5, *d* = 50 μm, *a* = 25 μm). (B) Relative error between round and square aperture. The maximum relative error between a square and a round aperture is 13,3% and occurs at *x = a/2*. At a distance *x = a* and beyond, approximating a square opening by a round opening overestimates the flow velocity by less than 2%. C) Plot of the isopotential lines for the velocity potential in normalized units X = 2x/a, Y = 2y/a for both round and square apertures. Both sets of isopotential lines, while significantly different near the aperture, become nearly identical a short distance away from the square aperture. It is useful to observe that, under the Hele-Shaw regime, the velocity potential is linked to the pressure field near an aperture by the proportionality constant $-{G^{2}}/{12\eta}$, as in Eq. (2).

3.1 Flow near a square monopole

In a 2D potential flow, the velocity at any point near a distribution of sources can be computed by a superposition of point sources. Drawing analogy with Coulomb’s Law in electrostatics, albeit in a 2D geometry instead of 3D, the general expression describing this superposition principle is given by the integral:

|  | (S1) |
| --- | --- |

where *Q* is the injected flow rate, *G* is the gap size between the probe and the surface, and *S* is the aperture surface. Notation refers to Fig. 1E.

Expressed in Cartesian coordinates, for a single square opening of surface *S = a^2^* whose center is located at the origin, this equation becomes :

| . | (S2) |
| --- | --- |

The above solution treats a square opening as a sum of point sources located between -a/2 and a/2.

All further analysis can be simplified by making (S2) dimensionless using the following groups: $\chi=\frac{2x}{a},\chi'=\frac{2x'}{a},\xi=\frac{2y}{a},\xi'=\frac{2y'}{a},$ which yields:

|  | (S3) |
| --- | --- |

The velocity potential can then be obtained by further integrating Eq. (S3) such that:

|  | (S4) |
| --- | --- |

It is of interest to note that Eq. (S4) also represents the expression of the dimensionless pressure underneath the probe as the pressure and velocity potential are directly proportional as described in Eq. (2). Analytical solutions exist for both Eq. (S3) and Eq. (S4), but offer little physical insight. Eq. (S4) is plotted in Fig. S3C and compared to the simple case of round apertures. To determine the position of the stagnation points, we are however only interested in the x-velocity component on the x-axis.

| , | (S5) |
| --- | --- |

whose solution is:

|  | (S6) |
| --- | --- |

This function represents the fluid velocity at a distance $\chi=2x/a$ from the center of a square opening perpendicular to one of its sides. Let’s rename this dimensionless function as:

|  | (S7) |
| --- | --- |

By comparison, the normalized velocity around a round opening can be obtained from Eq. (3) (with 2*r/a = χ*):

|  | (S8) |
| --- | --- |

The values of (S7) and (S8) are plotted against each other in Fig. S3A and S3B of the current paper, showing that when *x* > *a*, round and square openings have extremely similar flow profiles. Fig. S3C further confirms in 2D the fast convergence of both square and round apertures to the same potential lines when x>a.

3.2 Comparison between a square dipolar probe and a round dipolar probe

The total velocity around two square openings separated by a distance *d* at a distance *x* of the probe’s center is given by a superposition of these functions.

where . α is the aspiration/injection flow ratio.

The stagnation point on the axis of the MFP will be located at such that the following condition is preserved:

|  | (S9) |
| --- | --- |

The above equation is transcendental and has to be solved numerically. The *x*-component of the velocity profile, which is the function describing the normalized flow perpendicular to the distance between two apertures, has been plotted for circular and rectangular apertures (Fig. S3). A useful approximation can be made in the case where . We find that the solution approaches to the particular case of individual round openings, i.e. Eq. (7) in the main text.

3.4 Model corrections for square apertures

Square-aperture MFPs have been widely used, sometimes based on an arbitrary design, and other times due to microfabrication constraints. Certain applications, such as surface patterning, may also be better served by rectangular rather than round apertures.[^7^](#_ENREF_7) Yet, as seen above (Fig S3B), the difference between flow around a square MFP and a round -aperture MFP is negligible but to a distance closer than *a* outside the edge of the square apertures. We conclude that in most cases, efficient probe modeling can be carried out by using the round aperture model instead of the square one with minimal discrepancies. However, for MFPs when the half inter-aperture distance *d/2* is close to the square aperture size *a*, such as in the probe described in Fig. 3 (curve (d): *a* = 25 μm, *d* = 50 um) then the geometry of the aperture will influence the flow profile.

3.5 Model corrections for finite size apertures

When the aperture *a* becomes non negligible with respect to the inter aperture distance *d*, there will be distortions appearing due to the fact that the pressure and velocity profiles are different right below the apertures than everywhere else under the probe. Furthermore, in the real three-dimensional case, the flow under a MFP is supplied from the top wall into the gap, the approximation that the flow is purely two-dimensional is no longer valid right below the apertures. In fact, the hydraulic resistance of the device will be much less below the apertures due to the tubing extending vertically up and leaving room for the vertical jetting of fluid. This decreased resistance below the apertures will lead to a decreased resistance in the shortest flow path, i.e. along the x-axis from the inlet to the outlet, which has a characteristic length *d-a* instead of *d* in the case of the point source model. Therefore, the presence of apertures of non-negligible dimensions will cause the fluid injected under the probe to flow preferentially inward along this shortest path, with the effect of decreasing the outward flow rate. This results in the formation of a stagnation point closer than expected with point sources located at ± *d/2*. Yet, the variation on the position of the stagnation point should be negligible as long as *d >> a*. In Fig. 3A, we purposely model finite aperture sizes to test the predictive limit of the point source model in non-ideal cases. The relative errors on the position of the SP introduced by the finiteness of the aperture are respectively of 3-8% (*a/d* = 1/6) and 7-25% (*a/d* = 1/2). Fig. 3C-F shows the diffusion broadening and confinement area variations as a function of *α* and gap size *G* in two apertures MFP. All represent excellent matches between 2D analytical models and full 3D simulation. The larger discrepancies found pertain to curve (d) and in general to situations where *G* is no longer much smaller than the aperture size *a (G/a = 0.4)* and *a* no longer much smaller than distance *d* (*a/d* = 0.5). In these particular limiting cases, the Hele-Shaw condition is broken locally near the aperture. Eq. (13) correctly predicts the diffusion length behavior as the function of α and *G* which can be seen in Fig 3C-D. Calculations of confinement area have been explained in Fig. S2.

**4. Stability analysis of the HFC under the probe for varying flow rate ratios and gaps**

In Fig. 2, we observed both experimentally and numerically a disappearance of the hydrodynamic flow confinement under certain operating conditions, resulting in a decreased exposure concentration at the substrate’s surface below the probe. In the first case, Fig. 2A(8), the flow rate ratio is set to α = 8 while maintaining gap height at G = 10 μm for a probe of aperture ratio a/d = ½. In the second case, Fig. 2B(12), α was kept at 2.5 while G was set to 50 μm. The two losses of confinement can be explained quantitatively using the arguments below:

4.1 HFC disappearance with increasing flow rate ratio

As the flow rate ratio α is increased the stagnation point is found closer to the injection aperture. Past a critical α value, it is no longer defined as it resides under the injection aperture. Mathematically, this condition is expressed as *R < d/2 + a/2*. Using the expression of R found in Eq. (7), we get the critical α at which the SP will be defined as:

| $\alpha<1+\frac{2d}{a}.$ | (S10) |
| --- | --- |

In the case shown in Fig. 2, d/a = 2 and α < 5. For α > 5, we expect that the pattern will be lost underneath the probe. Using the reasoning provided above in section 3.5, stagnation points are always found closer to the injection aperture than theoretically predicted using a point source approximation, due to the decreased hydraulic resistance in the flow path directly between the two apertures. Thus, for systems where the ratio a/d is large (close to 1), it is likely that confinement will be lost at values smaller than expected in Eq. (S10). Nevertheless, the latter provides a useful simple criterion to ensure correct probe operation.

4.2 HFC disappearance with increasing gap

As the gap is increased between the probe mesa and the substrate, injected species will more predominantly flow to the aspiration outlet by a path closer to the probe mesa than the substrate. When G/d becomes large, no species is expected to reach the substrate before being recaptured by the aspiration aperture. $Re= {\rho\alpha Q_{inj}G}/{\pi ad\eta}\ll1$ (see section 6 below), but ${G^{2}}/{d^{2}}\gg1$. However, this behavior will occur only when the Hele-Shaw condition is broken. Within the Hele-Shaw condition, flow is expected to be uniform in the vertical (z direction). Therefore, the critical G at which this condition will be broken can be simply stated as :

| $\min\left(G=d , G=\frac{\pi ad\eta}{\rho\alpha Q}\right)$ | (S11) |
| --- | --- |

In the experimental case presented in Fig 2A panel 4, we get respectively from Eq. (S11) G = 50 μm and G = 1 mm. In most cases, unless the flow rate is very high, the constraint *G^2^<<d^2^ will be the limiting one. This condition explains why, when G =* 50 μm, the HFC confinement pattern is lost in Fig. 2B panel 12.

**5. Derivation of the Hele-Shaw flow profile for a probe moving at constant velocity**

When moving at constant velocity *U* above a static surface, a MFP will generate a Couette flow profile that will superpose to the flow profile arising from the injection and aspiration flow rates. In such circumstances, the Hele-Shaw flow profile, Eq. (1), has to be modified to account for new boundary conditions in the vertical direction, namely *v_H-S_*(x,y,0) = 0 and *v_H-S_* (x,y,G) = U. Thus the new Hele-Shaw profile respecting these conditions is now

|  | (S12) |
| --- | --- |

The height-averaged flow profile is thus given by

|  | (S13) |
| --- | --- |

Eq. (S13) can be made identical to Eq. (2) by performing the calculations in the reference frame of the Couette flow’s average velocity using the Galilean transformation

|  | (S14) |
| --- | --- |

In this reference frame, the probe’s velocity profile is identical to that of Eq. (5). The velocity profile can then be obtained in the reference frame of the moving probe by applying the transformation in Eq. (S14)

|  | (S15) |
| --- | --- |

where θ is the angle that $\vec{U}$ makes with the probe axis. This equation can be further simplified to Eq. (22) using the dimensionless groups described in Eq. (10).

**6. Low Reynolds Numbers Hele-Shaw Flow**

We provide here a derivation of the Hele-Shaw approximation from the full Navier-Stokes equation. This analysis yields explicit expression of the Reynolds number under the Hele-Shaw approximation as well as the geometrical constraints that must be obeyed to operate in this regime.

The motion of an incompressible flow of a Newtonian fluid is governed by the conservation of mass and momentum equations, given by (neglecting gravitational forces)

|  | (S16) |
| --- | --- |
|  | (S17) |

Where $\vec{V}\left( x,y,z \right)=(v_{x},v_{y},v_{z})$ is the velocity field, $p$is the pressure field, $\rho$and $\eta$ are, respectively, fluid density and dynamic viscosity. We render these governing equations dimensionless by introducing the dimensionless variables

|  | (S18) |
| --- | --- |

Where $d$ is the characteristic length scale in the $x,y$ directions, $G$ is the characteristic length scale in the $z$ direction, and $U$ is the characteristic velocity. The governing equations, known as the Navier-Stokes equations, in the dimensionless forms become (neglecting the unsteady term)

| $\frac{\rho UG}{\eta}\left(\frac{G}{d}\left[\hat{v}_{x}\frac{\partial\hat{v}_{x}}{\partial\hat{x}}+\hat{v}_{y}\frac{\partial\hat{v}_{x}}{\partial\hat{y}}\right]+\hat{v}_{z}\frac{\partial\hat{v}_{x}}{\partial\hat{z}}\right)=-\frac{\partial\hat{p}}{\partial\hat{x}}+\frac{G^{2}}{d^{2}}\left[\frac{\partial^{2}\hat{v}_{x}}{\partial\hat{x}^{2}}+\frac{\partial^{2}\hat{v}_{x}}{\partial\hat{y}^{2}}\right]+\frac{\partial^{2}\hat{v}_{x}}{\partial\hat{z}^{2}}$ $\frac{\rho UG}{\eta}\left(\frac{G}{d}\left[\hat{v}_{x}\frac{\partial\hat{v}_{y}}{\partial\hat{x}}+\hat{v}_{y}\frac{\partial\hat{v}_{y}}{\partial\hat{y}}\right]+\hat{v}_{z}\frac{\partial\hat{v}_{y}}{\partial\hat{z}}\right)=-\frac{\partial\hat{p}}{\partial\hat{y}}+\frac{G^{2}}{d^{2}}\left[\frac{\partial^{2}\hat{v}_{y}}{\partial\hat{x}^{2}}+\frac{\partial^{2}\hat{v}_{y}}{\partial\hat{y}^{2}}\right]+\frac{\partial^{2}\hat{v}_{y}}{\partial\hat{z}^{2}}$ $\frac{\rho UG}{\eta}\left(\frac{G}{d}\left[\hat{v}_{x}\frac{\partial\hat{v}_{z}}{\partial\hat{x}}+\hat{v}_{y}\frac{\partial\hat{v}_{z}}{\partial\hat{y}}\right]+\hat{v}_{z}\frac{\partial\hat{v}_{z}}{\partial\hat{z}}\right)=-\frac{d}{G}\frac{\partial\hat{p}}{\partial\hat{z}}+\frac{G^{2}}{d^{2}}\left[\frac{\partial^{2}\hat{v}_{z}}{\partial\hat{x}^{2}}+\frac{\partial^{2}\hat{v}_{z}}{\partial\hat{y}^{2}}\right]+\frac{\partial^{2}\hat{v}_{z}}{\partial\hat{z}^{2}}$ | (S19) |
| --- | --- |

When the gap $G$ between the plates relative to the distance between the apertures $d$ is sufficiently small, the terms on the order of ${G^{2}}/{d^{2}}$ can be omitted in front of the other terms. This conditions that ${G^{2}}/{d^{2}}\ll1$ is the first requirement for the Hele-Shaw approximation to be valid. We observe that, under this approximation, the effective Reynolds number in front of the dimensionless inertial term defined as $Re= {\rho UG^{2}}/{\eta d}$, as typical in flows under the lubrication approximation. Note that our analysis neglect the entrance effect in the proximity of the apertures, and the flow is approximated as purely in the $x,y$ directions. Setting $v_{z}=0$, and using the expression of Re derived above, we obtain

| $\mathrm{Re}\left[\hat{v}_{x}\frac{\partial\hat{v}_{x}}{\partial\hat{x}}+\hat{v}_{y}\frac{\partial\hat{v}_{x}}{\partial\hat{y}}\right]=-\frac{\partial\hat{p}}{\partial\hat{x}}+\frac{\partial^{2}\hat{v}_{x}}{\partial\hat{z}^{2}}$ $\mathrm{Re}\left[\hat{v}_{x}\frac{\partial\hat{v}_{y}}{\partial\hat{x}}+\hat{v}_{y}\frac{\partial\hat{v}_{y}}{\partial\hat{y}}\right]=-\frac{\partial\hat{p}}{\partial\hat{y}}+\frac{\partial^{2}\hat{v}_{y}}{\partial\hat{z}^{2}}$ $0=\frac{\partial\hat{p}}{\partial\hat{z}}$ | (S20) |
| --- | --- |

The last equation gives $\hat{p}=\hat{p}\left(\hat{x},\hat{y}\right)$ $.$ When $Re\ll1$, the inertial terms on the left hand side can be neglected in front of the pressure and viscous forces and the flow profile between the plates can therefore be described as a quasi two-dimensional flow by the Hele-Shaw approximation.[^22^](#_ENREF_22),[^23^](#_ENREF_23)

|  | (S21) |
| --- | --- |

with $z$ being the vertical distance above the bottom flat surface inside the probe where the no-slip boundary conditions are satisfied at $z=0$ and $z=G$. Note that the variables are converted back into their dimensional forms.

Water was used as a solvent with a density of 998.2 kg/m³ and dynamic viscosity *η* = 0.001 N·m/s (at 20°C). For the injection flow rate of $Q_{inj}$, the maximum velocity under the probe can be used to compute the highest possible Reynolds number. This highest velocity U is found right at the inner edge of the aspiration aperture, where $U={\alpha Q_{inj}}/{\pi aG}$. For the most stringent operating conditions used in this paper, $Q_{inj}=0.44 nL/s, G=50 \mu m, a=25 \mu m, d=50 \mu m,$ and $\alpha=4$, the maximum Reynolds number close to the aspiration aperture becomes ${Re}_{max}={\rho UG}/\eta={\rho\alpha Q_{inj}G}/{\pi ad\eta}=0.11$. In this particular extreme case, corresponding to the experimental results in Fig. 2B(9), G = d and the Hele-Shaw validity domain is violated. Thus Re <<1 is the second important requirement to fully validate the Hele-Shaw approximation. It is interesting to note that for all the probe geometries and operating conditions presented in this paper, the Hele-Shaw approximation breaks down before Re becomes larger than unity.
